# What happens after James Lind Alliance Priority Setting Partnerships? A qualitative study of contexts, processes and impacts

**DOI:** 10.1186/s40900-020-00210-9

**Published:** 2020-07-11

**Authors:** Kristina Staley, Sally Crowe, Joanna C. Crocker, Mary Madden, Trisha Greenhalgh

**Affiliations:** 1 TwoCan Associates, Ross on Wye, HR9 7DL UK; 2 Crowe Associates, Oxford, OX9 3LW UK; 3 Department of Primary Care Health Sciences, University of Oxford, Oxford, OX2 6GG UK; 4 Department of Health Sciences, University of York, York, YO10 5DD UK

**Keywords:** Patient and public involvement, James Lind Alliance, Priority setting partnerships, Research priorities

## Abstract

**Background:**

The James Lind Alliance (JLA) supports priority setting partnerships (PSPs) in which patients, carers and health professionals collaborate to identify a Top 10 list of research priorities. Few studies have examined how partnerships plan for the post-prioritisation phase, or how context and post-PSP processes influence the fortunes of priorities. This evaluation aimed to explore these questions.

**Methods:**

We selected a diverse sample of 20 interviewees who had knowledge of 25 PSPs. Thirteen interviewees had led a PSP, either from a university, patient organisation or charity. Three were patients who had taken part in a PSP workshop. Four others, three researchers and one funder, had worked with JLA PSP priorities to develop research proposals. We analysed the data thematically, exploring how success was understood and achieved.

**Results:**

The JLA PSPs had different histories, funding sources, goals and stakeholders. Whilst their focus was on generating priority research topics, PSPs’ wider impacts included enhanced status and greater confidence for individuals, as well as relationship-building and network strengthening for the organisations involved. To follow through on a Top 10, additional work was needed to refine broad priority topics into research questions and match them with appropriate funding sources. Commitment to post-PSP action from partners appeared to increase the chance that priority topics would be followed through to funded studies. Academic publications could alert researchers to a PSP’s outputs, but not all PSPs had the capacity to produce them. A Top 10 list potentially influences funding decisions through direct funding, themed calls or as a prompt in open calls. Influence on funders appears to depend on alignment between a priority and the funder’s remit, culture and values.

**Conclusion:**

The history and context of a JLA PSP have a major influence on its impact. Our findings suggest that there is no universal formula for success, but that greater resource and attention should be given to what happens after prioritisation. Further research is needed on what works best in what circumstances. Overall, we conclude that a wider cultural change in the research world is needed for JLA PSPs to achieve their goal of shaping the research agenda.

## Plain English summary

The James Lind Alliance (JLA) supports priority setting partnerships (PSPs) where patients, carers and health professionals work together to decide research priorities. Few studies have examined the impact of these priorities on research, or how impact was achieved. We explored impact through interviews with 20 people, who had been involved in a JLA PSP, or had worked with the Top 10 priorities. An Advisory Group helped to select the interviewees and questions asked. The project team looked at the interview transcripts for common themes and any differences.

JLA PSPs have unique histories, goals and people involved which affect the nature of their success. While they often lead to newly funded research, PSPs’ wider impacts include enhanced status and confidence for individuals, as well as relationship-building and network strengthening for the organisations involved. Additional work is needed post-PSP to change broad priorities into research questions. Journal articles alert researchers to the priorities, but not all PSPs can produce them. Research funders can respond to a priority through direct funding or calls for proposals. Influence on research funders appears to depend on a match between a priority and the funder’s remit, culture and values.

There is no universal formula for success, but greater resource and attention should be given to what happens after the Top 10. Further research is needed on what works best in what circumstances. Overall, we conclude that a deeper cultural change in the research world is needed for JLA PSPs to achieve their goal of shaping the research agenda.

## Background

The James Lind Alliance (JLA) supports priority setting partnerships (PSPs) in which patients, carers and health professionals collaborate to identify priority topics for new research, usually generating and publishing a Top 10 list [1]. Few studies have examined how partnerships plan for the post-prioritisation phase, or how context and post-PSP processes influence the fortunes of priority lists. We aimed to explore these questions through an evaluation of a range of JLA PSPs.

The JLA was established in 2003 with the goal of involving patients, carers and health professionals in shaping the research agenda [2, 3]. Its founders’ vision was that JLA partnerships would work together to review existing evidence on the treatment of a particular condition and identify and prioritise knowledge gaps. The scope of JLA PSPs now includes cause, diagnosis, social care and prognosis [4], and topics other than health conditions. It has become a popular and widely-used approach to research priority setting.

In 2013 the JLA became a partner organisation of the National Institute for Health Research (NIHR). The JLA Secretariat, made up of 2.5 full-time equivalent staff, is based at the Wessex Institute in Southampton, UK. It is responsible for maintaining the infrastructure and website, as well as contracting a network of ‘JLA Advisers’ who support the PSPs. The JLA Guidebook (now in its 8th edition) [5] details the structured JLA approach. PSPs can be initiated and led by anyone with a connection to the PSP’s topic (including a healthcare professional, patient, carer or researcher). Each PSP has a steering group which coordinates and implements the activity of the PSP. It includes patients, carers and clinicians and a JLA Adviser. Funding for PSP activity can be provided by organisations, charities and/or PSP partners with no commercial interest in the topic.

The recommended PSP process has developed over time. It now consists of eight steps after funding has been secured: form a steering group to lead the priority setting partnership; gather research uncertainties via a survey; categorise the survey responses to generate a longlist of uncertainties (40–70); prioritise this list in a second survey checking none have already been researched thus generating a short list (20–30); hold a final priority setting workshop to generate a Top 10 from the short list; publish both the short list and the Top 10; work with funders and researchers to influence their decisions; and monitor and evaluate the impact of the priorities [5–7]. The first six steps are well-established and highly structured. The last two have been added recently and are less well-developed. Whilst feedback from PSPs does filter into the JLA Guidebook as part of a regular review, there is no formal process for sharing learning between PSPs, especially on how to maximise impact from the Top 10 list.

The first JLA PSP on asthma was published in 2010 [8]. The approach has since been used by over 100 priority setting partnerships [4], mostly in the UK [9], but also in Canada [10, 11], Europe [12] and Africa [4]. The published literature on JLA PSPs is largely descriptive of the processes used by PSPs and their priorities [9]. To our knowledge, there has not been a formal audit of how many JLA Top 10 topics have received research funding, nor an in-depth evaluation of the impact of PSPs. This study aimed to highlight and begin to fill that gap.

Our research question had several parts. We first asked people who had participated in a JLA PSP or responded to a JLA PSP priority, what impacts they had identified. We then asked how these impacts had been influenced by the context and by processes *after* the PSP. We were interested in exploring not only the impacts on the commissioning or funding of new research, but also the impacts on the people and the partner organisations. We hoped the findings would be useful to past, present and future PSPs.

## Methods

### Management and governance

The study formed part of the Partnerships for Health, Wealth and Innovation theme in the NIHR Oxford Biomedical Research Centre (BRC) [13] and was conducted by two independent researchers, SC and KS. It was overseen by an Advisory Group of 11 people bringing the perspectives of patients, carers, PSP Leads from a charity or university context, health researchers, funders of research, experts in public involvement in research and a JLA Adviser. The Advisory Group helped to shape the project plans, agreeing the criteria used to choose interviewees (see Supplementary Material), and the broad topics explored in the interviews. They also reviewed the findings to help develop recommendations. The JLA Secretariat had no role in the project, other than identifying and accessing some interviewees.

The three patients/ carers on the Advisory Group were paid for their time and expenses. They were invited to join the Group because they had extensive experience of being involved in PSPs and/or were patient leaders in the field of public involvement in research. Along with all Advisory Group members, they were known to be reflective and constructively critical of their experiences of the JLA approach.

### Study design

Our approach was based on illuminative evaluation, which uses qualitative methods to explore the rationale, development, operations, achievements, and difficulties of an initiative [14]. It relies on engaging a wide range of stakeholders and capturing their perspective on what counts as impact, rather than judging the initiative against fixed external criteria.

### Sampling

We sought a diverse sample of interviewees who had organised or contributed to PSPs in a variety of roles and contexts (patients, carers and clinicians), or had worked with JLA PSP priorities to develop research projects or funding calls (researchers, staff from funding organisations). The final candidates were chosen to maximise the range of perspectives on the key questions identified with the Advisory Group. They fell into the categories listed in Table 1. A selection matrix made the questions and the criteria for selection transparent (see Supplementary Material). PSP interviewees were recruited with advice from the JLA secretariat and two JLA advisers. Open recruitment of researchers was via an advert on social media. Others were recruited via snowball sampling, through interviews with PSP Leads.

**Table 1:** Different categories of interviewee (the total adds up to more than 20 because some interviewees fell into more than one category)

Category of interviewee	Perspective provided by interviewees in this category	No. of interviewees
**PSP Lead** ***Charity / Patient organisation Funder***	**A person who led the work of a PSP as a key member of the Steering Group, and was employed by a charity or patient organisation that funds external researchers.**	**6**
**PSP Lead** ***Charity / Patient organisation Non-funder***	**A person who led the work of a PSP, as a key member of the Steering Group, and was employed by a charity that does not fund external researchers.**	**2**
**PSP Lead** ***Academic institution***	**A person who led the work of a PSP, as a key member of the Steering Group, and was employed by an academic institution such as a university or research centre.**	**4**
**PSP Lead** ***Clinical organisation***	**A person who led the work of a PSP, as a key member of the Steering Group, and was employed by a clinical organisation such as a hospital trust.**	**1**
**Public Funder**	**A manager from an organisation that allocates public funds to health research.**	**1**
**Clinician**	**A clinician who took part in a PSP and reflected on the impact on their practice.**	**2**
**Patient**	**A patient who took part in a PSP and reflected on the impact on their life and work.**	**3**
**Researcher**	**A researcher that did not take part in a PSP, but worked with a Top 10 priority list.**	**3**

### Interviews

Interviewees were invited by email and gave informed consent to take part. Telephone interviews were conducted in April–May 2019 and lasted 30–60 min. Semi-structured interview schedules were individualised to reflect the expertise and experience of each interviewee, but all interviews covered the following topics, where relevant:

- Who led the PSP – and who owns the outputs?
- What was the dissemination strategy?
- How have funders worked with the priority lists generated by this PSP?
- How have researchers worked with the priority lists?
- How has being involved in the PSP affected partner organisations?
- How has being involved in the PSP affected individuals?
- Examples of indirect impacts?

The interviews took the form of an exploratory conversation which allowed the interviewee to identify and explore topics important to them. All interviews were digitally recorded and transcribed. Recordings and pseudo-anonymised transcripts were stored on the Oxford University secure IT network. Whilst most interviewees were comfortable to be identified, one or two wished to be fully anonymised. Therefore some quotes have not been attributed to a particular PSP.

### Data analysis and synthesis

The researchers read all transcripts and agreed the broad themes, partly informed by discussions with the Advisory Group. Their analysis took the form of inductive thematic analysis [15], so that the themes were generated from the data up, identifying common themes – topics, ideas and patterns of meaning that came up repeatedly – and noting differences. Their interpretation was influenced by their standpoints as people working outside academia, with extensive experience of developing or supporting JLA PSPs, and of patient and public involvement in research. The findings have been published separately [16] and developed further for this article in discussion with TG, MM and JC.

## Results

We have categorised our findings in relation to the contextual and process factors that influenced outcomes and impacts. These are discussed in turn.

### Contextual factors influencing PSP impact

The JLA PSPs in our sample had been established by different groups for different reasons, and this was an important factor explaining their outputs and impacts. The PSP’s goals were influenced by the mission, capacity, practical constraints and influence of the lead organisation, a charity, patient organisation or academic institution. Some PSPs had set out to raise the profile of an underfunded research area. Others had aimed to broaden the scope or rank competing priorities in an area that was already popular or where priorities were contested. What counted as success therefore depended on the PSP’s starting point.

The wider political context to a PSP also influenced its process and outcomes. For example, in the *chronic* Lyme Disease PSP (a clinical condition whose origin and treatment is contested), the patient organisation was unable to recruit any clinical researchers for the Steering Group. The PSP Lead from Lyme Disease Action, reported that that some clinical researchers seemed to feel that the PSP *“shouldn’t even be happening”.* Seven years later, the Lyme Disease PSP priorities have yet to be addressed by any research in the UK, although some interest has been shown by other countries. Simply running a PSP cannot resolve historical or existing tensions in a research community, though it may help to move some debates forward.

### Post-PSP processes influencing impact

We have summarised these findings under seven broad themes related to post-PSP activity: planning for the end of the PSP, disseminating the Top 10, persuading funders and researchers to respond to the priorities, using JLA PSP research priorities to influence strategy and funding decisions, translating priority topics into research questions, evaluating the impact of a PSP, and the post-PSP impacts on people and organisations.

#### Planning for the end of the PSP

Following the recommendations in the current JLA guidebook [5], most PSP Leads in our sample had developed a comprehensive communications plan to raise awareness of their Top 10 research priorities and often the shortlist- considered at their final workshop. However, many PSP Leads questioned whether simply informing others of the outputs was sufficient to have impact. On reflection, they concluded they needed a more strategic approach to their post-PSP activity:

*“ … there's no point in identifying your Top 10, if you don’t have a plan of what you're going to do with them afterwards. We underestimated the work that’s required if you want to really make the best use of the results.”* - PSP Lead, Charity, Funder

Some reported having overlooked issues that could have been addressed in early planning, such as: considering how to achieve each of the PSP’s strategic goals; agreeing who will ‘own’ the PSP priority list; considering how the PSP will respond to non-research questions (for example, statements about deficiencies in current services); and capitalising on the skills, experience and networks that had been developed through the partnership.

Others described having made such plans. For example, the charity-led Mental Health in Children and Young People PSP (2018) aimed to raise professional and public awareness of mental health issues in young people as an additional outcome. They therefore included an All Party Parliamentary Group event in Parliament in their dissemination plan. The Type 2 Diabetes PSP (2017), led by Diabetes UK, built alliances within their organisation to facilitate dissemination of priorities (via the communications department) and address topics relating to service improvement outside the remit of the PSP (via the quality department). They also engaged external partners at the beginning with a view to developing joint funding calls at the end.

#### Disseminating the top 10 priority list

Typically, all or most partner organisations involved in a PSP helped with dissemination of the outputs. Large organisations leading PSPs usually had communications teams to provide expert advice and resource for this. Target audiences included patients, carers and the public, research funders, policy makers, health professionals, lay media and researchers. Interviewees felt that it was important to convey details of how Top 10 lists are generated. They considered that few other researchers, journal editors or academic reviewers fully understood or valued the rigour of the process:

*“I don’t think they realise that the process has been quite long, very involved, and involved hundreds of people in different ways.” -* Researcher

Successful dissemination strategies included: a press release summary of the Top 10 embargoed until launch day; a launch event e.g. at a scientific conference; a plain English summary for patients and carers; an academic journal article; social media activity e.g. releasing one priority per day over 10 days, or a guest blog; hosting a web page; using email lists of PSP survey participants; giving talks to patient, clinical and scientific groups; a funding organisation alerting current and past grant holders; using the channels and networks of partner organisations e.g. their newsletters; and promoting findings by word of mouth.

Many PSPs involved patients, carers and clinicians in their dissemination activity, based on the rationale that this gave the messages greater credibility and authenticity.

Some PSPs in our sample had successfully published their findings in academic journals, hoping to reach researchers in the field. However, some had experience of having papers rejected, because the identified priorities were not considered to be ‘new knowledge’. Some interviewees suspected this was because the JLA approach was no longer seen as novel. In some cases, the PSP team lacked the expertise to write academic articles and navigate the publication process. They saw this as a missed opportunity.

*“ … all we could do was link to the article on our website … a clinician, if they see that the link is to a patient website, they’re not going to take it so seriously, but if we had had an [academic] paper, that would have been an anchor, more trustworthy from their point of view.*” - PSP Lead, Charity, Non-funder

One interviewee expressed uncertainty about the status of priority setting as a topic for academic publication, especially in relation to the narrow formats deemed appropriate for many clinical journals:

*“I don’t think any journal’s going to be interested in our Top 10 … It doesn’t seem scientific, there’s no hypothesis. There isn’t really a research question … it's more social science isn't it?”* - PSP Lead, Academic Institution

PSP Leads reported varied experience in achieving national news coverage. Several had been told that their priority list was not “newsworthy”. They felt that science journalists were more interested in reporting scientific ‘breakthroughs’ rather than how the research question came about. The PSPs that had received mainstream media coverage had made links with reporters with personal or family experience of the condition.

Some interviewees concluded that the main audience for Top 10s was researchers and more effort was needed to reach them. While some had produced a lengthy report for their patient/carer community, they were doubtful of its value and concluded that a short report would be sufficient.

“*It’s this thing about charities constantly churning out PDF reports and you need to make sure you’re asking yourself ‘What is the point, right? Like who is reading this?’”* PSP Lead, Charity, Funder

#### Persuading others to respond to the top 10

The key stakeholders that PSPs aimed to influence were funders, researchers and donors. In order to get their priorities addressed by external funders, PSP partners first needed to identify whom to influence and then target activities towards these individuals or organisations. However, this sometimes proved challenging – for example, if the funding body did not recognise the JLA process or value its outputs.

Interviewees from external funding bodies emphasised that JLA priority lists were only one of many influences on their portfolio, which typically took account of the organisation’s remit and mission, its accountabilities to a wide range of stakeholders, prevailing scientific and policy priorities, and the opinions of review panels – as well as the amount of funding available: *“Sometimes it’s a question of who is the best funder of this research … and often that’s not us*.” – Funder.

Involving external funders directly by inviting them to Steering Group meetings or workshops, or involving them indirectly (e.g. by using their premises for meetings) was not found to be sufficient by PSP Leads to influence those funders’ decisions. At best, it helped to start a relationship. Greater value seemed to come from PSP partners working closely with funders in the post-PSP phase – for example to develop themed calls (see below).

PSP Leads emphasised the value of using influencing skills at this stage, which might require involving different people from the team that ran the PSP. Whilst ideally a PSP would include individuals with all the relevant skills, several PSPs in our sample either had not planned for this, or lacked resources. PSP Steering Groups sometimes felt exhausted by the time they “*got their PSP over the finishing line*” (Advisory Group Member), and because funding had ended, key individuals had little time or energy for follow-up.

Loss of momentum at the post-PSP stage was a particular problem for PSPs which lacked a strong sponsor such as patient organisation or charity. Cellulitis, for example, is a potential complication of numerous other conditions. No organisation existed for cellulitis itself. As the PSP Lead explained, this meant that the Cellulitis PSP, “*… didn’t have a critical mass of people willing to keep it up in the air.”* This was in contrast to the same interviewee’s experience of leading the Eczema PSP which had the strength of the Eczema Society behind it.

In terms of influencing researchers, our interviews suggested that researchers tended to use a Top 10 priority to strengthen the case for a study they already planned to do. Some said the JLA PSP gave them confidence and motivation to persist with applying for a grant on a prioritised topic. We heard no reports of researchers changing their research plans to pick up a PSP priority, nor of researchers stopping pursuing an area of interest because it was not on a priority list.

*"It [the Top 10] was great for including in these sections of the grant and fellowship applications about what is the impact of your research going to be, and what’s the need for this research.* - Researcher

Where an academic institution had led a PSP, the partnership was keen to emphasise that that the Top 10 list was not ‘owned’ by them, but meant for all researchers in their field. In one such example, working in partnership with a charity was found to be an effective means of reaching and potentially influencing others. In this case, the charity’s CEO provided the missing skills and links with the wider research community, as well as acting as an ‘honest broker’ from "*an organisation that doesn’t have any skin in the game"* [PSP Lead, Charity, Funder].

Finally, in relation to donations, some of the patient interviewees felt that a Top 10 could provide an effective focus for fundraising by patients and carers. Some charity-led PSPs reported that the JLA process had indeed helped to convince their donors that specific research projects were worthy of their support:

*If you can show with clarity that you’re representing the priorities of your people … that is enormously powerful and it gives you enormous confidence in terms of going out there and speaking about what you do, especially with donors …* -PSP Lead, Charity, Funder

#### Using the priorities to influence funder strategy and decisions

When a PSP was led by an organisation that funds research, there were several ways in which priorities were used to influence its own funding decisions and those of other funders: integrating these into organisational research strategy; directly funding research studies; sharing the list with other related funders and seeking their input to joint programmes of research; putting out themed calls for research proposals; integrating priority topics into open calls for funding applications; or using the priorities as a criterion when judging the importance of research proposals:

*“[The JLA PSP has] given us justification to prioritise and rationalise with the research community, because people with [this condition] have told us what they want us to do. What was also important was making sure our Board of Trustees knew about those ten priorities, because now we've got a Board that understands and wants us to report against them*.” - PSP Lead, Charity, Funder

Some funders had successfully worked together to develop research calls on shared priorities e.g. Parkinson’s UK joined forces with Marie Curie and other charity PSPs where a question about managing incontinence featured in their Top 10. Several of the PSP Leads interviewed from charities, were also exploring the possibility of their organisation putting out a joint call with the NIHR.

Some Top 10-themed research calls generated a poor response leading to the view that the research community lacked the skills, capacity or willingness to address the topic. Charity funders have taken additional steps to bring researchers together to carry out foundational work to develop new areas of research. For example, the MS Society worked with research groups to develop a plan for a clinical trial that was ultimately funded by a charity in the USA. In another PSP, prevention and minimising risk emerged as a priority topic, but capacity in that area was limited, so the charity organised an international workshop to develop recommendations for follow-up work.

Whilst one or two interviewees felt that all funded research should reflect priority topics, others felt that patients, carers and clinicians involved in the process may lack insight about potentially valuable research avenues: “*There will be some basic science questions that were never going to be brought up in a PSP which could be key to scientific and medical progress” –* PSP Lead, Academic Institution. None of the charities interviewed for this study had made the decision to *only* fund JLA PSP priorities*.* In open funding calls, JLA priority lists were one of several criteria used by reviewers to assess applications.

Some interviewees raised concerns that researchers might use a JLA PSP priority to ‘game the system’. For example, by squeezing their preferred research idea into a broad umbrella priority topic, or claiming that their proposal addresses a JLA PSP without justifying this in depth. Some charity funders assumed that the involvement of patients and carers in the review process provided an adequate check. However, they were uncertain how any grant reviewers assessed the researchers’ claims in practice. Many interviewees also commented that addressing a Top 10 topic did not mean that researchers could then ignore the patient voice in the subsequent research process. On the contrary, as one PSP Lead described, “*the JLA is the beginning of an involvement process [with patients]” -* PSP Lead, Charity, Funder.

#### Translating a priority topic into a research project

Translating a Top 10 priority into a research project has three steps: (1) mapping which aspects of the topic remain unanswered; (2) developing a focused research question from the broad priority (where necessary); and (3) designing a project to address the question. Many of the PSPs in this sample had hosted workshops with researchers to carry out this translation work. To our surprise, few had involved patients and carers, though several had involved clinicians. Yet our data suggested that patient and carer involvement at this stage could reduce the risk of priorities being misinterpreted and translated back into conventional clinician and researcher framings.

“*Question eight on our [Top 10] list is about how parenting styles affect treatment outcomes for young people with mental health problems. And it seemed when we were talking to funders that they got the wrong end of the stick about this … A lot of the research that is out there is about how parents who have a mental health problem affect their children. That’s not what this question is getting at all … ”* PSP Lead, Mental Health in Children and Young People

One PSP Lead described a situation in which a funder had not worked with anyone from the PSP, and had developed a poor-quality call as a result:

“*The funder did end up putting out a call on one of the questions, but they didn’t speak to anyone beforehand … and the call was so badly worded that no one could put a research proposal together to answer it. It just went unfunded*.” - PSP Lead, Charity, Funder

In another example, the NIHR put out a commissioning call in response to the priority topic ‘the management of continence problems’ from the Childhood Disability PSP. The call asked for a survey of current NHS practice in this area. Parents of disabled children who worked with researchers to respond to the call pointed out that the proposed survey would fail to identify any measures taken by parents and families themselves. They included this omission in their successful application, and two of the parents became co-investigators on the funded study.

#### Evaluating the difference JLA PSPs are making to research

Interviewees from research charities reported assessing the impact of their PSP by monitoring how many grant applications addressed a priority topic. Some did this by including a tick-box in their application form and suggested that public funders such as NIHR follow suit. Many PSP Leads had kept track of which priorities had been addressed by funded research. For example, the Mild to Moderate Hearing Loss PSP identified nine such projects, including studentships, fellowships, systematic reviews and a feasibility study. However, it is not helpful to judge the impact of JLA PSPs simply in terms of how much funding is allocated to their priorities, since some research topics can be addressed with modestly-funded studies, while others require an expensive clinical trial.

Some PSPs had been able to share details of research progress with their patient, carer and researcher communities. For example, the Tinnitus PSP (2012) produced a research update report for its members 5 years later [17]. Two of the patient interviewees said they found it hard to find the information they would like about progress. It was unclear who had responsibility for keeping such information up to date and whether it should be held by the JLA Secretariat, PSPs, researchers or funders.

#### Transforming: the impact of PSP involvement on individuals and organisations

The experience of going through the JLA process was reported to have a profound and mostly positive impact on the people and organisations who took part. Patients and carers said they felt they had made a unique and important contribution to their PSPs. They described gains in knowledge and confidence which had affected their own lives and work, and had led to an appetite for subsequent involvement in research and campaigning, although not all had been able to find such opportunities:

*“It was the first time that I realised that patients should and could have a say in the whole healthcare ecosystem. They weren’t just victims or the ‘done to’ but that they could positively impact what’s going on.”* - Patient participant in a PSP

Clinicians who had been involved in PSPs reported that the experience had changed their clinical practice, making them more aware of patients’ concerns and highlighting aspects of healthcare that need improving. In some cases it enhanced their own visibility, status and credibility amongst peers. They also felt more able and confident to involve patients and carers in their work, and expanded their ‘involvement’ networks:

*“The PSP brought me into contact with a patient charity … I heard stories that I would never have heard otherwise, about how doctors relate to patients, and how the words used are important such as giving people fear, hope or being realistic.”*

-Clinician lead, Inherited Anaemias PSP

Similar transformational changes were described by organisations that had taken part in a PSP. The experience was reported to have enhanced their status and credibility, for example, one charity described being seen as “the” patient voice for their condition in the UK after their PSP. Others reported a change in their organisations’ cultures and values towards more patient-centred and collaborative ways of working. PSPs often left a legacy of infrastructure, policies and projects to support ongoing patient involvement. Newly built or strengthened networks encouraged partner organisations to continue working together on areas of common interest, and a shift to a more collaborative way of working led to new approaches to working with funded researchers.

Finally some PSPs reported success in transforming national policy. The Tinnitus PSP identified an absence of clinical guidelines for tinnitus in children and drew on the PSP networks to convene an inter-professional group to draft new guidance. The Canadian Dementia PSP directly influenced the Canadian government’s dementia strategy to highlight the issue of stigma and the need for early treatment [18].

## Discussion

Even within this small scale qualitative study of relatively few PSPs we have begun to reveal a rich and complex picture of what counts as outcomes and impacts of JLA PSPs which goes far beyond simply funding research. The context for each PSP is hugely significant. The starting point of the PSP, what it aims to achieve, the individuals involved, the organisations that lead it – all shape the process and outcomes, making it difficult to draw out general conclusions about ‘how to succeed’ and broadening the definition of what success looks like.

Some PSPs, particularly in the early days, understood the production of a Top 10 to be an end in itself, but over time, a need to plan strategically and adequately resource an additional stage of post-PSP work has become more apparent. The difficulty described by interviewees in getting the outputs of JLA PSPs published in academic journals is concerning. Changes in the publishing landscape in recent years such as the emergence of niche journals like Research Involvement and Engagement and the rise of open access publishing (requiring a publication fee) may also have influenced whether and where JLA PSP findings appeared in the academic literature.

The evolving strength of the *partnership* itself and its capacity to influence others seems as important as the narrow *product* of a JLA PSP (the Top 10 list). Our findings did not reveal a simple relationship between generating priorities and people acting on them. Rather, priorities became transformed into funded projects when patients, clinicians, researchers, funders and policymakers became aligned behind them, sometimes in indirect and non-linear ways. This reflects Carol Weiss’s classic theoretical work in the sense that policy, in this case research policy, is rarely directly knowledge-driven. More often policy priorities emerge through a kind of mutual enlightenment, in which different stakeholders, through repeated interaction over time, come to understand and value each other’s perspectives [19]. There are different and sometimes competing understandings of the concepts of research ‘use’ or ‘impact’. To communicate more effectively in wider research policy making and funding systems, PSPs need to understand how funders process evidence of priorities and the environments in which they and researchers operate [20].

### Lessons for improving the impact of priority setting partnerships

Whilst it is impossible to provide a ‘blueprint’ for a successful PSP, we offer some broad recommendations for improving the process and maximising impacts in Table 2. Since this project was completed, SC and KS have discussed the findings with a wide range of stakeholders in two separate workshops: The JLA Advisory Group meeting and The Impact Coffee Club hosted by NIHR. The feedback was synthesised into a report for the JLA community [21].

**Table 2:** Suggestions for improving the impact of James Lind Alliance Priority Setting Partnerships

**For the JLA**	
➣ Extend JLA guidance to cover impact-oriented activity beyond producing a Top 10 list	
➣Ensure that JLA Advisors are trained and resourced to support planning such activity	
**For JLA PSPs**	
*At the planning stage:*	
➣ Consider who will own the Top 10 list once it has been produced, and how the list might be taken forward	
➣ Plan early for wide dissemination and associated networking and lobbying activity	
➣ Ensure that resources are allocated for such ‘post Top 10’ activities	
*During and after the Top 10 list has been produced:*	
➣ Make strategic use of patients, carers, clinicians and researchers in promoting the Top 10 list	
➣ Work with funders after dissemination of the Top 10 list, recognising that (depending on culture, remit and priorities) they may not believe it is their responsibility to address the priorities identified	
➣ Work with clinicians, patients and carers to develop specific research questions and projects from the Top 10 list	
➣ Do foundational work to build researchers’ capacity and willingness to respond (e.g. interdisciplinary workshops to promote collective thinking)	
**For research funders, and researchers** ➣ Recognise that patient, carer and clinician input has an important place in research priority setting, and promote this culture in your organisation	
➣ Improve your understanding of the JLA process and its goals, including how a Top 10 list is produced and what additional data may be available from a JLA PSP	
➣ Recognise that whilst some JLA PSP priority topics may not be new, the way that patients, carers and clinicians frame their questions may bring a different perspective, requiring a novel methodological response and closer dialogue between researchers and patients, carers and clinicians	
➣ Recognise that not all research topics and questions should be framed as PICO (population-intervention-comparison-outcome) and addressed in clinical trials	
➣ Provide training for reviewers of funding applications (including patient and public reviewers) about the JLA PSP process, and support a systematic approach (e.g. with criteria) for reviewers to judge whether a research proposal genuinely reflects a prioritised question	
**For academic journals**	
➣ Designate and promote a specific publication genre (‘Research Priority Setting’) for PSPs (and others) to publish their findings	
➣ Encourage peer reviewers of such publications to be aware of the steps set out in the JLA guidebook	
➣ Tag such publications with standard key words such as ‘research priority setting’ to make them easily retrievable	

The recommendations from these workshops for the JLA included [21]: creating new opportunities for shared learning across PSPs, both past and present, in addition to the current website advice; assessing a potential partnership’s readiness to address issues of impact as part of the initial appraisal of PSPs; creating a library of JLA PSP results; and, providing more information to PSPs about the research funding landscape and how best to work with funders. It was also suggested that research funders and researchers improve their understanding of how a Top 10 is generated and make better use of the survey data that underpins each priority when developing new research questions. Funders could usefully share their experience of assessing whether grant applications genuinely address a JLA priority to develop best practice guidance in this area.

### Strengths and limitations of this study

To our knowledge, this is the first qualitative study of how context and post-PSP processes influence outputs and impacts. A systematic literature review was not conducted to confirm this. The scope of the project was limited by the modest budget and timescale. Our use of exploratory, semi-structured interviews allowed participants to make sense of complex and emergent processes and comment reflexively on what had occurred after their PSP had finished. The lack of existing quantitative data on impact collected across all PSPs or direct observation of actual PSP practices means that these retrospective accounts by interviewees could not be subject to comparison or fully put into context. Despite these limitations, however, we believe the study has begun to scope the issue of JLA PSPs’ impact and usefully identified areas for future research and evaluation.

## Conclusion

This illuminative evaluation of JLA PSPs has highlighted the complex links between processes post-PSP. We have identified ways in which the JLA Secretariat and the PSPs themselves might maximise their influence. We have also shown a vital need for parallel commitment from other parts of the wider research system to adapt how they view and respond to JLA PSP priorities. Whilst much progress has already been made, much deeper and broader cultural change is required to ensure that the goal of delivering research that is relevant and useful to the end-users is truly achieved.

## Supplementary information

**Additional file 1: Supplementary material 1.** Topics of enquiry / Matrix of criteria used to select interviewees.

## Data Availability

The datasets used and/or analysed during the current study are available from the corresponding author on reasonable request.

## Abbreviations

IT: Information technology
JLA: James Lind Alliance
PPI: Patient and public involvement
PSP: Priority Setting Partnership
UK: United Kingdom
USA: United States of America
