# Supplementary material for: What happens after James Lind Alliance Priority Setting Partnerships? A qualitative study of contexts, processes and impacts
Source: Res Involv Engagem. 2020 Jul 11;6:41. doi: 10.1186/s40900-020-00210-9 (PMC7353437; doi:10.1186/s40900-020-00210-9)
Supplement: Supplementary file 1 — Additional file 1: Supplementary material 1. Topics of enquiry / Matrix of criteria used to select interviewees. [file 40900_2020_210_MOESM1_ESM.docx]

**Supplementary material 1**: **Topics of enquiry /** **Matrix of criteria used to select interviewees**

In the table below the numbered statements indicate the broad areas of enquiry covered in the interviews. The bullet lists beneath each question indicate the criteria used to select interviewees and the specific questions that were asked of them. We were looking for interviewees who could talk about specific kinds of experiences. This matrix was developed by the researchers (SC and KS) with advice from the Advisory Group and the JLA Secretariat to agree the range of experiences that would be explored through the interviews.

Each potential interviewee was assessed across all the criteria. The final list of interviewees was chosen so that all criteria were met by the combination of people in the sample. The interview questions were designed so that interviewees could raise new topics relating to outputs and impact in the post-PSP phase, so conversations were not limited by these criteria.

| **Criteria used to select interviewees** | |
| --- | --- |
| 1. **Who led the PSP and who owns the outputs? How does this influence what happens next?** | |
| **Type of interviewee** | **Questions asked** |
| PSP Lead from a charity that funds research | Whether/ how priorities have influenced internal research strategy or funding decisions? |
| PSP Lead from a patient organisation that doesn’t fund research | Whether/how have they influenced funders? |
| PSP Lead from an NIHR organisation with funding for research e.g. a Biomedical Research Centre | Whether/how priorities have influenced internal research strategy or funding decisions? |
| PSP Lead from an umbrella organisation of multiple funders | Whether/ how have they influenced members’ funding decisions? |
| PSP Lead from an clinical organisation | Whether/ how have they influenced funders? |
| PSP Lead from a co-led PSP model whereby several organisations have contributed funds and ‘own’ the outputs | Whether/ how have they influenced members’ funding decisions? |
|  | |
| 1. **How have priorities been disseminated/ shared with funders/ researchers/other stakeholders?** | |
| **Type of interviewee** | **Questions asked** |
| All PSP Leads | What was their dissemination strategy? |
| Interviewees who worked with a priority | How did they hear about the Top 10? |
| PSP Leads that engaged funders as part of the PSP process e.g. funders on Steering Group | What impact did this have? |
| PSP Leads who have experienced barriers to dissemination | Where has publication of a Top 10 been blocked and why? |
| PSP Leans who have used grassroots activity to raises awareness of JLA priorities | What impact did this have? |
|  | |
| 1. **How have funders worked with the priorities? And to what end?** | |
| **Type of interviewee** | **Questions asked** |
| PSP Leads from Charities/NIHR organisations that have allocated funding to priority topic | Whether/ how do JLA priorities influence funding decisions, research strategy etc? |
| Staff member from a Charity/NIHR organisation that developed call for proposals based on a priority | How did this develop and what was the outcome? |
| PSP leads from Charities that set up working group to develop projects from priorities | How did these develop and what was the outcome? |
| PSP Leads from Charities/ NIHR organisations where funders and researchers collaborated on translating priorities | How did these develop and what was the outcome? |
| PSPs Leads from Charities that have collaborated on common priority areas to identify the highest priority issues affected broad range of conditions | How did this develop and what was the outcome? |
|  | |
| 1. **How have researchers worked with priorities? & to what end?** | |
| **Type of interviewee** | **Questions asked** |
| Researchers/ research groups that have developed a project based on a priority topic | How did this come about and what were the outcomes? |
| PSP Leads from PSPs where no researchers were working on the priorities | Whether/ how did they address issues of capacity? |
| Senior researchers | What influence has JLA PSPs had on their field as a whole – has the research landscape changed in any way? |
|  | |
| 1. **How has taking part in a PSP affected the partner organisations post-PSP?** | |
| **Type of interviewee** | **Questions asked** |
| All PSP Leads from charities/ NIHR organisations | Whether/ what was impact on organisational culture? |
| PSPs with partnerships of multiple organisations | What impact did the experience have post-PSP? |
|  | |
| **6. How has it affected the individuals who took part in the PSP?** | |
| **Type of interviewee** | **Questions asked** |
| Patients, Carers and PSP Leads | What has been the impact on them personally and professionally? |
|  | |
| **7. How have the other outputs of JLA PSPs been used?** | |
| **Type of interviewee** | **Questions asked** |
| PSP Leads from organisations known to have worked with the known unknowns (the questions asked by survey respondents that had already been answered by research) | How did this come about and what were the outcomes? |
| PSP Leads from organisations known to have worked with the out of scope questions (the questions asked by survey respondents that were not directly answerable by research e.g. about health professional training or access to services). | How did this come about and what were the outcomes? |
|  | |
